# Using anti-DEI policies to dismantle education: A new front in a really old battle

**DOI:** 10.1371/journal.pbio.3003256

**Published:** 2025-06-20

**Authors:** Cissy J. Ballen, Terry McGlynn, Kelly R. Zamudio

**Affiliations:** 1 Department of Biological Sciences, Auburn University, Auburn, Alabama, United States of America; 2 Department of Biology, California State University Dominguez Hills, Carson, California, United States of America; 3 Department of Integrative Biology, The University of Texas, Austin, Texas, United States of America

## Abstract

Educational equity faces renewed threats across higher education in the United States. This Perspective addresses what can be done by life science instructors and researchers to prioritize equitable, evidence-based teaching for all.

## Teaser

Educational equity faces renewed threats across higher education in the United States, undermining learning for all. What can the community of life science instructors and researchers do to prioritize equitable, evidence-based teaching?

A basic principle of public education is to ensure equal access and opportunity for students to learn. In the United States, the Supreme Court unanimously recognized this in Brown versus the Board of Education (1954), when they terminated legal segregation in schools and wrote that public education “is the very foundation of good citizenship” [1]. This principle of educational equity is the goal to which the entire enterprise aspires (Box 1).

**Box 1. Defining educational equity.**

Historical and ongoing debates among researchers and policymakers have considered what constitutes an equitable education, what it looks like in practice across variable classrooms, and how to attain it [2]. We broadly define this term as providing all students with what they need to succeed in a learning environment. This includes equity in: access to educational resources (or input, e.g., funding and qualified educators), opportunities for learning (or process, e.g., pedagogy), and learning outcomes (or output, often measured through assessments) [3].

As educators, our policies and practices have been built on the principle that all students are worthy of access and opportunity. Students majoring in life sciences follow one of the countless career paths: they may pursue a career as a healthcare provider, a microbiologist, a science educator, or as something outside of science altogether. What unites these trajectories is the most basic principle in life sciences education: that each student should receive the support and resources they need to fulfill their own potential. Thus, the recent shift in how diversity, equity, and inclusion (DEI) efforts are portrayed—as exclusionary [4]—and the use of that argument to justify the dismantling of the US national education system are fundamentally part of the same strategy. The recent anti-DEI policies that disproportionately impact undergraduate education may seem like a new battlefield, but the battle is an old one, one that dates back well before 1954.

Recent attempts to dismantle the US Department of Education, as well as the termination of over a thousand research awards by the National Science Foundation supporting science, technology, engineering, and mathematics (STEM) education research and practice, harm educational equity and target members of society with the least access to educational opportunity. These cuts discriminate against some, but will worsen learning for all.

Faced with this onslaught, it is easy for individuals to feel powerless. What can one biology instructor do when a state law restricts what they can teach? What can one researcher do when their work is defunded or politically targeted? The damage done to educational equity has been dramatic, yet there is a lot that cannot be taken from the community of instructors and researchers who hold educational equity as a guiding principle.

First, we call on life science departments and institutions to look inward and more fully support teaching and learning efforts. For too long, universities have relied on external sources to develop and sustain solutions to our greatest educational challenges; it is time that we all make those innovations in teaching part of our core mission. Hiring discipline-based education researchers, who use data-informed reflection to interrogate student data, advances our knowledge of what works in STEM teaching and maximizes learning outcomes for all students. This knowledge has led to several tangible recommendations that have changed how we teach in higher education. For example, we know from large-scale research efforts in undergraduate STEM classes that active learning strategies, such as small group work, mini-lectures, and in-class activities, disrupt educational inequities that systematically disadvantaged capable but academically underprepared students [5], while at the same time leading to more effective learning for all students [6]. In biology education specifically, scholars have offered guidance on the use of inclusive language to foster belonging in the classroom [7], emphasized the importance of representing contemporary biologists in curricula so students can envision their ‘possible selves’ in science [8], shown the power of field-based research in increasing student persistence [9], and demonstrated how engaging students with authentic data builds scientific literacy [10]. Researchers have also developed approaches for navigating sensitive topics at the intersection of biology and society—such as human health disparities or climate change—in ways that promote respectful dialogue and critical engagement [11]. Valuing these examples and other evidence-based equitable teaching efforts in tenure and promotion cases is one strategy to make inclusive teaching a central priority. But going one step beyond requires investing in the study of these strategies to understand them across institutional context, and analyzing the direct links between the innovation, the mechanism, and the potentially disparate learning and affective outcomes among students. These changes will be a big shift for US R1 universities, which have historically focused on disciplinary research as their foundational goal. We argue that the cost of this shift is offset by the unique opportunity to make educational equity an integral part of a new academic landscape, with far reaching positive societal impact.

Second, we urge institutions to also look outward, and engage beyond their own walls, to support and inspire the extended community of biology educators. When institutions innovate and improve their teaching practices, these changes have ripple effects on the people and institutions within their sphere of influence. When we adopt evidence-based biology teaching practices that are explicitly designed to promote educational equity, this provides models for students that will eventually teach in middle school, high school, and in other 2-year and 4-year institutions, and vastly increases our societal impact. Mutual partnerships between 2-year and 4-year institutions, teacher development programs and collaborations with school districts, extend the educational mission to include professional development of biology educators to maximize educational opportunities for all.

While current policies pose broad challenges to science education, collective action offers a meaningful path forward. Fortunately, applying evidence-based teaching strategies that support educational equity is “just good teaching” [12], and good teaching cannot be taken away from those who practice it.

## Abbreviations

DEI: diversity, equity, and inclusion
STEM: science, technology, engineering, and mathematics
